# 3-(2,4-Dichloro­phen­oxy)-1-(4-meth­oxy­phen­yl)-4-(3-nitro­phen­yl)azetidin-2-one

**DOI:** 10.1107/S1600536810052645

**Published:** 2010-12-18

**Authors:** Mehmet Akkurt, Sevim Türktekin, Aliasghar Jarrahpour, Seid Ali Torabi Badrabady, Orhan Büyükgüngör

**Affiliations:** a Department of Physics, Faculty of Sciences, Erciyes University, 38039 Kayseri, Turkey; b Department of Chemistry, College of Sciences, Shiraz University, 71454 Shiraz, Iran; c Department of Physics, Faculty of Arts and Sciences, Ondokuz Mayıs University, 55139 Samsun, Turkey

## Abstract

In the title compound, C_22_H_16_Cl_2_N_2_O_5_, the nearly planar four-membered β-lactam ring [maximum deviations of 0.011 (2) for the N atom] makes dihedral angles of 68.34 (13), 83.04 (13) and 3.37 (13)° with the dichloro-, nitro- and meth­oxy­phenyl rings, respectively. The crystal structure is stabilized by C—H⋯O hydrogen-bond inter­actions. In addition, a π–π stacking inter­action [centroid–centroid distance = 3.6622 (12) Å] is observed between the β-lactam and nitro­phenyl rings.

## Related literature

For general background on β-lactams, see: Alcaide & Almendros (2001 ▶); Alcaide *et al.* (2007 ▶); Banik *et al.* (2004 ▶); Jarrahpour & Ebrahimi (2010 ▶); Jarrahpour & Zarei (2009 ▶); Jarrahpour *et al.* (2007 ▶); Turos *et al.* (2005 ▶); Vatmurge *et al.* (2008 ▶).
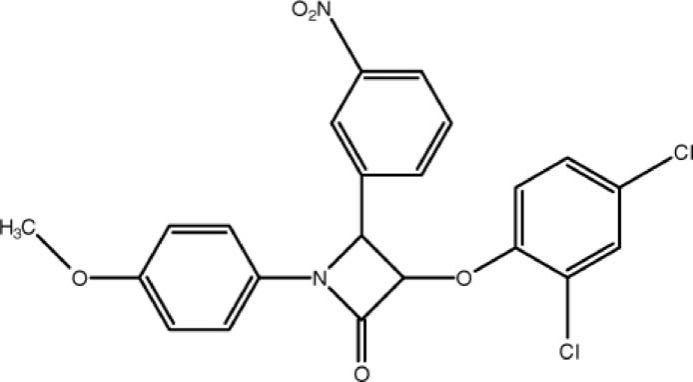


## Experimental

### 

#### Crystal data

C_22_H_16_Cl_2_N_2_O_5_
*M* *_r_* = 459.27
Orthorhombic, 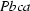

*a* = 9.0406 (2) Å
*b* = 17.8177 (5) Å
*c* = 25.9964 (6) Å
*V* = 4187.57 (18) Å^3^
*Z* = 8
Mo *K*α radiation
μ = 0.35 mm^−1^
*T* = 296 K
0.57 × 0.41 × 0.28 mm

#### Data collection

Stoe IPDS 2 diffractometer
Absorption correction: integration (*X-RED32*; Stoe & Cie, 2002 ▶) *T*_min_ = 0.826, *T*_max_ = 0.909
38140 measured reflections
4199 independent reflections
3153 reflections with *I* > 2σ(*I*)
*R*_int_ = 0.075

#### Refinement

*R*[*F* ^2^ > 2σ(*F* ^2^)] = 0.047
*wR*(*F* ^2^) = 0.129
*S* = 1.05
4199 reflections
282 parameters
H-atom parameters constrained
Δρ_max_ = 0.23 e Å^−3^
Δρ_min_ = −0.34 e Å^−3^

### 

Data collection: *X-AREA* (Stoe & Cie, 2002 ▶); cell refinement: *X-AREA*; data reduction: *X-RED32* (Stoe & Cie, 2002 ▶); program(s) used to solve structure: *SIR97* (Altomare *et al.*, 1999 ▶); program(s) used to refine structure: *SHELXL97* (Sheldrick, 2008 ▶); molecular graphics: *ORTEP-3* (Farrugia, 1997 ▶); software used to prepare material for publication: *WinGX* (Farrugia, 1999 ▶).

## Supplementary Material

Crystal structure: contains datablocks global, I. DOI: 10.1107/S1600536810052645/om2390sup1.cif

Structure factors: contains datablocks I. DOI: 10.1107/S1600536810052645/om2390Isup2.hkl

Additional supplementary materials:  crystallographic information; 3D view; checkCIF report

## Figures and Tables

**Table 1:** Hydrogen-bond geometry (Å, °)

*D*—H⋯*A*	*D*—H	H⋯*A*	*D*⋯*A*	*D*—H⋯*A*
C3—H3⋯O3^i^	0.93	2.60	3.360 (3)	140
C7—H7⋯O2^ii^	0.98	2.56	3.287 (3)	131
C17—H17⋯O2	0.93	2.55	3.148 (3)	123
C18—H18⋯O5^iii^	0.93	2.48	3.382 (3)	165

Symmetry codes: (i) 
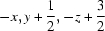
; (ii) 
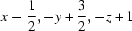
; (iii) 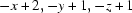.

**Fig. 1.**
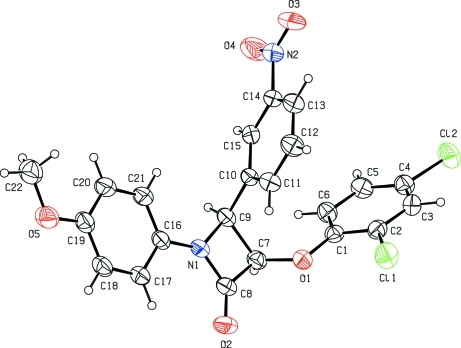
View of the title compound with the atom numbering scheme. Displacement ellipsoids for non-H atoms are drawn at the 30% probability level.

**Fig. 2.**
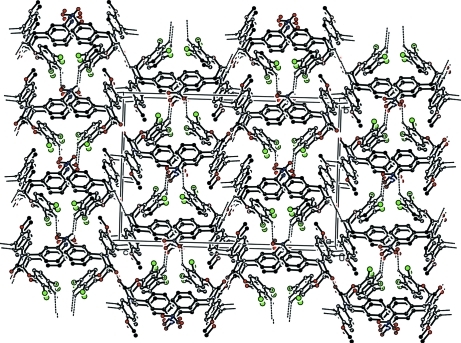
View of the packing and hydrogen bonding interactions down the a axis. Non-hydrogen bonding H atoms are omitted for clarity.

## supplementary crystallographic information

### Comment

Azetidin-2-one or β-Lactam ring system is a usual skeleton structure of antibiotic drugs like penicillins, cephalosporins, carbapenems, nocardicins and monobactams (Vatmurge *et al.*, 2008), that account for 50% of total antibiotics of the world (Alcaide *et al.*, 2001), and play an important role in the fight against pathogenic bacteria. β-Lactams possess very biological activity such as antibacterial (Jarrahpour *et al.*, 2009), anticancer (Banik *et al.*, 2004), antifungal (Jarrahpour *et al.*, 2010), antiviral (Jarrahpour *et al.*, 2007), and anti-MRSA (Turos *et al.*, 2005). β-Lactams can be used as a starting materials for the synthesis of other biological and medicinal substances such as α- and β-amino acids, β-amino alcohols, amino sugars, alkaloids, heterocycles, and taxoids and show the importance of β-Lactam ring as synthetic intermediates (Alcaide *et al.*, 2007).

In the title compound, (Fig. 1), the β-lactam ring (N1/C7–C9) is nearly planar, with maximum deviations of -0.011 (2) for N1 and 0.010 (2) Å for C8. The O1—C7—C8—O2, C20–C19—O5—C22, O3—N2—C14—C13, O4—N2—C14—C13, Cl1—C2—C3—C4 and Cl2—C4—C3—C2 torsion angles are -60.3 (3), 6.6 (4), 0.3 (4), 179.6 (3), 179.51 (17) and 177.69 (17) °. The dihedral angles between the ring planes are listed in Table 2.

In the crystal structure, molecules are linked by intermolecular C—H···O hydrogen-bond interactions (Table 1 and Fig. 2). Furthermore, a π-π stacking interaction [centroid-centroid distance = 3.6622 (12) Å] is present in the structure, between the β-lactam ring (N1/C7–C9) and the benzene ring (C10–C15) attached to the nitro group.

### Experimental

To a solution of *N*-(3-nitrobenzylidene)-4-methoxybenzenamine (1.0 eq.) in dry CH_2_Cl_2_ was added 2,4-dichlorophenoxy acetic acid (1.5 eq.), triethylamine (3.0 eq.), *p*-toluenesulfonyl chloride (1.5 eq.) and stirred at room temperature. After 10 h, the mixture was washed with 1*M* HCl (20 ml), saturated sodium bicarbonate solution (20 ml), brine (20 ml), dried over sodium sulfate and the solvent was evaporated to give the crude product as a gray precipitate which was then purified by recrystallization from EtOAc. (Yield 80%). [mp: 434–436 K]. IR (KBr, cm^-1^): 1763 (CO β-Lactam); ^1^H-NMR (250 MHz, CDCl_3_) δ (p.p.m): 3.67 (OCH_3_, s, 3H), 5.44 (H-9, d, 1H, *J* = 5.0 Hz), 5.49 (H-7, d, 1H, *J* = 5.0 Hz), 6.73–8.21 (ArH, m, 11H); ^13^C-NMR (62.9 MHz, CDCl_3_) δ (p.p.m): 55.49 (OMe), 60.38 (C-9), 81.63 (C-7), 114.67–156.98 (aromatic carbons), 161.30 (CO β-Lactam). Analysis calculated for C_22_H_16_Cl_2_N_2_O_5_: C 57.53, H 3.51, N 6.10%. found: C 57.42, H 3.45, N 6.23%.

### Refinement

H atoms attached to C atoms were placed at calculated positions and were treated as riding on their parent atoms with C—H = 0.93 (aromatic), 0.96 (methyl) or 0.98 Å (methine), and with *U*_iso_(H) = 1.5*U*_eq_(C) for methyl and *U*_iso_(H) = 1.2*U*_eq_(C) for aromatic, methine.

### Crystal data

**Table:**

C_22_H_16_Cl_2_N_2_O_5_	*F*(000) = 1888
*M**_r_* = 459.27	*D*_x_ = 1.457 Mg m^−^^3^
Orthorhombic, *P**b**c**a*	Mo *K*α radiation, λ = 0.71073 Å
Hall symbol: -P 2ac 2ab	Cell parameters from 34508 reflections
*a* = 9.0406 (2) Å	θ = 1.4–26.8°
*b* = 17.8177 (5) Å	µ = 0.35 mm^−^^1^
*c* = 25.9964 (6) Å	*T* = 296 K
*V* = 4187.57 (18) Å^3^	Prism, colourless
*Z* = 8	0.57 × 0.41 × 0.28 mm

### Data collection

**Table:**

Stoe IPDS 2 diffractometer	4199 independent reflections
Radiation source: sealed X-ray tube, 12 x 0.4 mm long-fine focus	3153 reflections with *I* > 2σ(*I*)
plane graphite	*R*_int_ = 0.075
Detector resolution: 6.67 pixels mm^-1^	θ_max_ = 26.2°, θ_min_ = 1.6°
ω scans	*h* = −11→11
Absorption correction: integration (*X-RED32*; Stoe & Cie, 2002)	*k* = −22→22
*T*_min_ = 0.826, *T*_max_ = 0.909	*l* = −32→32
38140 measured reflections	

### Refinement

**Table:**

Refinement on *F*^2^	Secondary atom site location: difference Fourier map
Least-squares matrix: full	Hydrogen site location: inferred from neighbouring sites
*R*[*F*^2^ > 2σ(*F*^2^)] = 0.047	H-atom parameters constrained
*wR*(*F*^2^) = 0.129	*w* = 1/[σ^2^(*F*_o_^2^) + (0.070*P*)^2^ + 0.3877*P*] where *P* = (*F*_o_^2^ + 2*F*_c_^2^)/3
*S* = 1.05	(Δ/σ)_max_ < 0.001
4199 reflections	Δρ_max_ = 0.23 e Å^−^^3^
282 parameters	Δρ_min_ = −0.34 e Å^−^^3^
0 restraints	Extinction correction: *SHELXL97* (Sheldrick, 2008), FC^*^=KFC[1+0.001XFC^2^Λ^3^/SIN(2Θ)]^-1/4^
Primary atom site location: structure-invariant direct methods	Extinction coefficient: 0.0019 (5)

### Special details

**Table:**

Geometry. Bond distances, angles *etc*. have been calculated using the rounded fractional coordinates. All su's are estimated from the variances of the (full) variance-covariance matrix. The cell e.s.d.'s are taken into account in the estimation of distances, angles and torsion angles
Refinement. Refinement on *F*^2^ for ALL reflections except those flagged by the user for potential systematic errors. Weighted *R*-factors *wR* and all goodnesses of fit *S* are based on *F*^2^, conventional *R*-factors *R* are based on *F*, with *F* set to zero for negative *F*^2^. The observed criterion of *F*^2^ > σ(*F*^2^) is used only for calculating -*R*-factor-obs *etc*. and is not relevant to the choice of reflections for refinement. *R*-factors based on *F*^2^ are statistically about twice as large as those based on *F*, and *R*-factors based on ALL data will be even larger.

### Fractional atomic coordinates and isotropic or equivalent isotropic displacement parameters (Å^2^)

**Table:**

	*x*	*y*	*z*	*U*_iso_*/*U*_eq_	
Cl1	0.21262 (8)	0.86082 (4)	0.66036 (3)	0.0868 (3)	
Cl2	−0.34474 (8)	0.78256 (5)	0.70616 (3)	0.0919 (3)	
O1	0.19780 (15)	0.74409 (8)	0.58582 (5)	0.0565 (5)	
O2	0.4456 (2)	0.72877 (11)	0.50535 (6)	0.0855 (7)	
O3	0.0887 (3)	0.49540 (13)	0.78799 (7)	0.1238 (12)	
O4	0.0058 (3)	0.45763 (14)	0.71618 (9)	0.1210 (10)	
O5	0.9134 (2)	0.44061 (12)	0.55088 (7)	0.0914 (7)	
N1	0.4083 (2)	0.61733 (10)	0.55214 (6)	0.0564 (6)	
N2	0.0844 (3)	0.49729 (13)	0.74126 (8)	0.0856 (9)	
C1	0.0686 (2)	0.74787 (11)	0.61302 (7)	0.0504 (6)	
C2	0.0615 (2)	0.80367 (12)	0.65049 (7)	0.0557 (6)	
C3	−0.0640 (3)	0.81334 (13)	0.67948 (8)	0.0631 (7)	
C4	−0.1839 (3)	0.76777 (13)	0.67092 (8)	0.0625 (7)	
C5	−0.1788 (2)	0.71157 (14)	0.63466 (8)	0.0633 (7)	
C6	−0.0516 (2)	0.70191 (13)	0.60600 (7)	0.0582 (7)	
C7	0.2181 (2)	0.68260 (13)	0.55226 (7)	0.0568 (7)	
C8	0.3747 (3)	0.68511 (13)	0.53065 (7)	0.0609 (7)	
C9	0.2637 (2)	0.60649 (12)	0.57725 (7)	0.0535 (6)	
C10	0.2670 (2)	0.60270 (11)	0.63547 (6)	0.0501 (6)	
C11	0.3591 (2)	0.64886 (13)	0.66385 (7)	0.0596 (7)	
C12	0.3587 (3)	0.64666 (15)	0.71726 (8)	0.0728 (9)	
C13	0.2688 (3)	0.59739 (15)	0.74266 (8)	0.0732 (9)	
C14	0.1795 (3)	0.55169 (13)	0.71435 (8)	0.0629 (7)	
C15	0.1755 (2)	0.55356 (12)	0.66085 (7)	0.0565 (6)	
C16	0.5354 (2)	0.57158 (12)	0.55186 (7)	0.0540 (6)	
C17	0.6591 (2)	0.59165 (13)	0.52329 (8)	0.0614 (7)	
C18	0.7819 (2)	0.54645 (14)	0.52407 (8)	0.0658 (8)	
C19	0.7845 (3)	0.48143 (15)	0.55296 (8)	0.0657 (8)	
C20	0.6621 (3)	0.46155 (15)	0.58117 (9)	0.0750 (8)	
C21	0.5384 (3)	0.50650 (14)	0.58026 (8)	0.0685 (8)	
C22	0.9267 (3)	0.37785 (19)	0.58396 (10)	0.0931 (11)	
H3	−0.06780	0.85040	0.70460	0.0760*	
H5	−0.26000	0.68050	0.62950	0.0760*	
H6	−0.04730	0.66380	0.58160	0.0700*	
H7	0.14100	0.67790	0.52590	0.0680*	
H9	0.20930	0.56420	0.56230	0.0640*	
H11	0.42220	0.68190	0.64690	0.0720*	
H12	0.41990	0.67880	0.73580	0.0870*	
H13	0.26850	0.59510	0.77840	0.0880*	
H15	0.11210	0.52220	0.64270	0.0680*	
H17	0.65860	0.63540	0.50380	0.0740*	
H18	0.86460	0.55980	0.50490	0.0790*	
H20	0.66290	0.41790	0.60080	0.0900*	
H21	0.45550	0.49260	0.59920	0.0820*	
H22A	0.84910	0.34270	0.57660	0.1110*	
H22B	0.91910	0.39410	0.61910	0.1110*	
H22C	1.02090	0.35420	0.57860	0.1110*	

### Atomic displacement parameters (Å^2^)

**Table:**

	*U*^11^	*U*^22^	*U*^33^	*U*^12^	*U*^13^	*U*^23^
Cl1	0.0833 (5)	0.0746 (4)	0.1024 (5)	−0.0248 (3)	0.0095 (3)	−0.0215 (3)
Cl2	0.0737 (4)	0.1076 (6)	0.0944 (5)	0.0119 (4)	0.0284 (3)	−0.0017 (4)
O1	0.0547 (8)	0.0589 (9)	0.0560 (7)	0.0000 (7)	0.0057 (6)	0.0002 (6)
O2	0.0927 (13)	0.0846 (12)	0.0791 (10)	0.0041 (10)	0.0313 (9)	0.0292 (9)
O3	0.194 (3)	0.1049 (16)	0.0724 (11)	−0.0032 (17)	0.0507 (14)	0.0294 (10)
O4	0.161 (2)	0.0992 (16)	0.1027 (15)	−0.0574 (17)	0.0489 (15)	−0.0058 (13)
O5	0.0724 (11)	0.1072 (15)	0.0946 (12)	0.0299 (11)	0.0292 (9)	0.0270 (11)
N1	0.0581 (10)	0.0616 (11)	0.0496 (8)	0.0027 (9)	0.0153 (7)	0.0052 (7)
N2	0.119 (2)	0.0620 (13)	0.0757 (14)	0.0005 (14)	0.0386 (13)	0.0087 (10)
C1	0.0516 (11)	0.0512 (11)	0.0485 (9)	0.0041 (9)	−0.0012 (8)	0.0065 (8)
C2	0.0615 (12)	0.0478 (11)	0.0579 (10)	−0.0003 (9)	−0.0014 (9)	0.0013 (8)
C3	0.0738 (15)	0.0539 (12)	0.0615 (11)	0.0062 (11)	0.0063 (10)	−0.0056 (9)
C4	0.0605 (13)	0.0668 (14)	0.0603 (11)	0.0068 (11)	0.0071 (9)	0.0041 (10)
C5	0.0553 (12)	0.0725 (14)	0.0621 (11)	−0.0060 (11)	0.0001 (9)	0.0017 (10)
C6	0.0594 (12)	0.0628 (13)	0.0523 (10)	−0.0027 (10)	−0.0015 (9)	−0.0060 (9)
C7	0.0609 (12)	0.0654 (13)	0.0442 (9)	0.0040 (10)	0.0027 (8)	0.0022 (9)
C8	0.0684 (13)	0.0693 (14)	0.0451 (9)	0.0016 (11)	0.0107 (9)	0.0056 (9)
C9	0.0538 (11)	0.0610 (13)	0.0457 (9)	−0.0024 (10)	0.0092 (8)	−0.0028 (8)
C10	0.0512 (11)	0.0529 (11)	0.0461 (9)	0.0053 (9)	0.0111 (8)	0.0042 (8)
C11	0.0569 (12)	0.0695 (14)	0.0524 (10)	−0.0043 (10)	0.0026 (9)	0.0046 (9)
C12	0.0780 (16)	0.0854 (17)	0.0551 (11)	−0.0040 (13)	−0.0052 (11)	−0.0028 (11)
C13	0.0942 (18)	0.0748 (16)	0.0507 (10)	0.0088 (14)	0.0085 (11)	0.0055 (11)
C14	0.0803 (15)	0.0532 (12)	0.0553 (11)	0.0092 (11)	0.0237 (10)	0.0102 (9)
C15	0.0627 (12)	0.0493 (11)	0.0576 (10)	0.0027 (10)	0.0134 (9)	−0.0015 (9)
C16	0.0550 (11)	0.0611 (12)	0.0460 (9)	0.0001 (10)	0.0110 (8)	−0.0033 (8)
C17	0.0656 (13)	0.0640 (13)	0.0547 (11)	−0.0022 (11)	0.0173 (9)	0.0018 (9)
C18	0.0588 (13)	0.0769 (16)	0.0618 (11)	−0.0014 (12)	0.0203 (10)	−0.0004 (11)
C19	0.0609 (13)	0.0759 (16)	0.0604 (11)	0.0090 (11)	0.0145 (10)	−0.0013 (10)
C20	0.0758 (15)	0.0774 (16)	0.0719 (13)	0.0126 (13)	0.0243 (11)	0.0179 (12)
C21	0.0666 (14)	0.0698 (15)	0.0691 (13)	0.0067 (12)	0.0269 (11)	0.0123 (11)
C22	0.086 (2)	0.110 (2)	0.0834 (16)	0.0294 (17)	0.0107 (14)	0.0117 (15)

### Geometric parameters (Å, °)

**Table:**

Cl1—C2	1.723 (2)	C12—C13	1.367 (4)
Cl2—C4	1.739 (3)	C13—C14	1.363 (3)
O1—C1	1.367 (2)	C14—C15	1.392 (3)
O1—C7	1.413 (3)	C16—C17	1.389 (3)
O2—C8	1.204 (3)	C16—C21	1.375 (3)
O3—N2	1.216 (3)	C17—C18	1.372 (3)
O4—N2	1.196 (3)	C18—C19	1.381 (3)
O5—C19	1.375 (3)	C19—C20	1.374 (4)
O5—C22	1.416 (4)	C20—C21	1.376 (4)
N1—C8	1.365 (3)	C3—H3	0.9300
N1—C9	1.474 (3)	C5—H5	0.9300
N1—C16	1.409 (3)	C6—H6	0.9300
N2—C14	1.472 (3)	C7—H7	0.9800
C1—C2	1.393 (3)	C9—H9	0.9800
C1—C6	1.373 (3)	C11—H11	0.9300
C2—C3	1.373 (3)	C12—H12	0.9300
C3—C4	1.373 (4)	C13—H13	0.9300
C4—C5	1.376 (3)	C15—H15	0.9300
C5—C6	1.381 (3)	C17—H17	0.9300
C7—C8	1.524 (3)	C18—H18	0.9300
C7—C9	1.559 (3)	C20—H20	0.9300
C9—C10	1.515 (2)	C21—H21	0.9300
C10—C11	1.384 (3)	C22—H22A	0.9600
C10—C15	1.373 (3)	C22—H22B	0.9600
C11—C12	1.389 (3)	C22—H22C	0.9600
			
Cl1···O1	2.8458 (16)	C15···Cl1^vii^	3.580 (2)
Cl1···O4^i^	3.400 (3)	C16···C11	3.593 (3)
Cl1···C15^i^	3.580 (2)	C17···O2	3.148 (3)
Cl2···C12^ii^	3.624 (3)	C18···O5^ix^	3.382 (3)
Cl2···O4^iii^	3.452 (3)	C21···C10	3.319 (3)
Cl2···C3^iv^	3.615 (2)	C3···H20^i^	2.9100
Cl2···C12^iv^	3.635 (3)	C4···H12^iv^	3.0500
Cl2···H12^ii^	2.9200	C6···H7	2.7500
Cl2···H22B^i^	3.0900	C7···H11	3.0800
O1···Cl1	2.8458 (16)	C7···H6	2.5400
O1···O2	3.077 (2)	C8···H17	2.8000
O1···N1	3.081 (2)	C8···H11	3.0500
O1···C11	3.020 (2)	C9···H21	2.7300
O2···C7^v^	3.287 (3)	C9···H6	2.9900
O2···O1	3.077 (2)	C10···H21	2.7600
O2···C17	3.148 (3)	C18···H6^xiii^	3.0000
O2···C1^v^	3.298 (2)	C18···H9^xiv^	2.9900
O2···C6^v^	3.147 (3)	C20···H22A	2.7100
O3···C3^vi^	3.360 (3)	C20···H22B	2.8000
O3···C12^iv^	3.407 (4)	C22···H20	2.5300
O4···Cl1^vii^	3.400 (3)	C22···H7^xiv^	3.0900
O4···Cl2^viii^	3.452 (3)	H3···O3^xi^	2.6000
O5···C18^ix^	3.382 (3)	H3···O4^xi^	2.8600
O1···H11	2.8000	H6···C7	2.5400
O2···H7^v^	2.5600	H6···C9	2.9900
O2···H17	2.5500	H6···C18^ii^	3.0000
O3···H13	2.4200	H6···H7	2.2500
O3···H3^vi^	2.6000	H7···C6	2.7500
O4···H15	2.4300	H7···H6	2.2500
O4···H22B^ii^	2.8700	H7···O2^x^	2.5600
O4···H3^vi^	2.8600	H7···C22^xiv^	3.0900
O5···H18^ix^	2.4800	H9···H15	2.3900
N1···O1	3.081 (2)	H9···C18^xiv^	2.9900
N2···C13^iv^	3.391 (4)	H11···O1	2.8000
N1···H11	2.7200	H11···N1	2.7200
C1···C10	3.201 (3)	H11···C7	3.0800
C1···C11	3.429 (3)	H11···C8	3.0500
C1···O2^x^	3.298 (2)	H12···Cl2^xiii^	2.9200
C3···O3^xi^	3.360 (3)	H12···C4^xii^	3.0500
C3···Cl2^xii^	3.615 (2)	H13···O3	2.4200
C6···C10	3.465 (3)	H15···O4	2.4300
C6···C9	3.402 (3)	H15···H9	2.3900
C6···O2^x^	3.147 (3)	H17···O2	2.5500
C7···O2^x^	3.287 (3)	H17···C8	2.8000
C8···C11	3.525 (3)	H18···O5^ix^	2.4800
C9···C6	3.402 (3)	H20···C22	2.5300
C10···C21	3.319 (3)	H20···H22A	2.2400
C10···C6	3.465 (3)	H20···H22B	2.4000
C10···C1	3.201 (3)	H20···C3^vii^	2.9100
C11···O1	3.020 (2)	H21···C9	2.7300
C11···C16	3.593 (3)	H21···C10	2.7600
C11···C8	3.525 (3)	H22A···C20	2.7100
C11···C1	3.429 (3)	H22A···H20	2.2400
C12···O3^xii^	3.407 (4)	H22B···O4^xiii^	2.8700
C12···Cl2^xii^	3.635 (3)	H22B···C20	2.8000
C12···Cl2^xiii^	3.624 (3)	H22B···H20	2.4000
C13···N2^xii^	3.391 (4)	H22B···Cl2^vii^	3.0900
			
C1—O1—C7	117.95 (15)	C16—C17—C18	119.5 (2)
C19—O5—C22	117.8 (2)	C17—C18—C19	121.0 (2)
C8—N1—C9	95.73 (17)	O5—C19—C18	115.9 (2)
C8—N1—C16	133.74 (19)	O5—C19—C20	124.6 (2)
C9—N1—C16	130.53 (17)	C18—C19—C20	119.5 (2)
O3—N2—O4	123.2 (3)	C19—C20—C21	119.7 (2)
O3—N2—C14	118.3 (2)	C16—C21—C20	121.1 (2)
O4—N2—C14	118.5 (2)	C2—C3—H3	120.00
O1—C1—C2	115.86 (16)	C4—C3—H3	120.00
O1—C1—C6	125.32 (17)	C4—C5—H5	120.00
C2—C1—C6	118.82 (17)	C6—C5—H5	120.00
Cl1—C2—C1	119.29 (14)	C1—C6—H6	120.00
Cl1—C2—C3	119.96 (16)	C5—C6—H6	120.00
C1—C2—C3	120.76 (19)	O1—C7—H7	114.00
C2—C3—C4	119.3 (2)	C8—C7—H7	114.00
Cl2—C4—C3	119.04 (17)	C9—C7—H7	114.00
Cl2—C4—C5	119.95 (19)	N1—C9—H9	112.00
C3—C4—C5	121.0 (2)	C7—C9—H9	112.00
C4—C5—C6	119.2 (2)	C10—C9—H9	112.00
C1—C6—C5	120.9 (2)	C10—C11—H11	120.00
O1—C7—C8	109.01 (16)	C12—C11—H11	120.00
O1—C7—C9	116.85 (15)	C11—C12—H12	120.00
C8—C7—C9	86.19 (15)	C13—C12—H12	120.00
O2—C8—N1	132.6 (2)	C12—C13—H13	121.00
O2—C8—C7	135.7 (2)	C14—C13—H13	121.00
N1—C8—C7	91.71 (17)	C10—C15—H15	121.00
N1—C9—C7	86.33 (14)	C14—C15—H15	121.00
N1—C9—C10	115.51 (15)	C16—C17—H17	120.00
C7—C9—C10	117.40 (16)	C18—C17—H17	120.00
C9—C10—C11	121.20 (17)	C17—C18—H18	120.00
C9—C10—C15	119.76 (16)	C19—C18—H18	120.00
C11—C10—C15	119.03 (16)	C19—C20—H20	120.00
C10—C11—C12	121.0 (2)	C21—C20—H20	120.00
C11—C12—C13	120.2 (2)	C16—C21—H21	120.00
C12—C13—C14	118.4 (2)	C20—C21—H21	119.00
N2—C14—C13	118.87 (19)	O5—C22—H22A	109.00
N2—C14—C15	118.4 (2)	O5—C22—H22B	109.00
C13—C14—C15	122.7 (2)	O5—C22—H22C	109.00
C10—C15—C14	118.67 (19)	H22A—C22—H22B	110.00
N1—C16—C17	120.69 (19)	H22A—C22—H22C	109.00
N1—C16—C21	120.09 (18)	H22B—C22—H22C	109.00
C17—C16—C21	119.2 (2)		
			
C7—O1—C1—C2	171.82 (16)	C4—C5—C6—C1	−0.5 (3)
C7—O1—C1—C6	−8.5 (3)	C8—C7—C9—C10	115.53 (17)
C1—O1—C7—C9	−78.4 (2)	C9—C7—C8—O2	−177.4 (3)
C1—O1—C7—C8	−173.88 (16)	O1—C7—C9—N1	−110.79 (16)
C22—O5—C19—C18	173.6 (2)	O1—C7—C8—O2	−60.3 (3)
C22—O5—C19—C20	−6.6 (4)	C9—C7—C8—N1	1.51 (15)
C16—N1—C8—O2	−2.3 (4)	O1—C7—C8—N1	118.62 (16)
C8—N1—C16—C21	176.3 (2)	O1—C7—C9—C10	6.1 (2)
C8—N1—C16—C17	−3.4 (3)	C8—C7—C9—N1	−1.40 (14)
C9—N1—C16—C17	177.12 (19)	C7—C9—C10—C11	−60.5 (2)
C16—N1—C9—C10	62.5 (3)	C7—C9—C10—C15	118.4 (2)
C8—N1—C9—C7	1.56 (15)	N1—C9—C10—C11	39.2 (3)
C9—N1—C8—C7	−1.60 (15)	N1—C9—C10—C15	−141.92 (19)
C16—N1—C8—C7	178.8 (2)	C9—C10—C11—C12	178.2 (2)
C9—N1—C8—O2	177.4 (2)	C11—C10—C15—C14	−0.4 (3)
C9—N1—C16—C21	−3.3 (3)	C15—C10—C11—C12	−0.8 (3)
C16—N1—C9—C7	−178.78 (19)	C9—C10—C15—C14	−179.34 (19)
C8—N1—C9—C10	−117.14 (19)	C10—C11—C12—C13	1.4 (4)
O4—N2—C14—C15	−1.1 (4)	C11—C12—C13—C14	−0.7 (4)
O3—N2—C14—C15	179.6 (2)	C12—C13—C14—C15	−0.5 (4)
O4—N2—C14—C13	179.6 (3)	C12—C13—C14—N2	178.8 (2)
O3—N2—C14—C13	0.3 (4)	C13—C14—C15—C10	1.0 (4)
C6—C1—C2—C3	−0.9 (3)	N2—C14—C15—C10	−178.2 (2)
C2—C1—C6—C5	1.4 (3)	N1—C16—C21—C20	−179.0 (2)
O1—C1—C6—C5	−178.31 (19)	C17—C16—C21—C20	0.6 (3)
C6—C1—C2—Cl1	179.03 (15)	N1—C16—C17—C18	179.40 (19)
O1—C1—C2—C3	178.88 (18)	C21—C16—C17—C18	−0.2 (3)
O1—C1—C2—Cl1	−1.2 (2)	C16—C17—C18—C19	−0.2 (3)
C1—C2—C3—C4	−0.6 (3)	C17—C18—C19—C20	0.3 (3)
Cl1—C2—C3—C4	179.51 (17)	C17—C18—C19—O5	−179.9 (2)
C2—C3—C4—C5	1.6 (3)	O5—C19—C20—C21	−179.7 (2)
C2—C3—C4—Cl2	−177.69 (17)	C18—C19—C20—C21	0.1 (4)
C3—C4—C5—C6	−1.0 (3)	C19—C20—C21—C16	−0.6 (4)
Cl2—C4—C5—C6	178.21 (17)		

Symmetry codes: (i) −*x*+1/2, *y*+1/2, *z*; (ii) *x*−1, *y*, *z*; (iii) −*x*−1/2, *y*+1/2, *z*; (iv) *x*−1/2, *y*, −*z*+3/2; (v) *x*+1/2, −*y*+3/2, −*z*+1; (vi) −*x*, *y*−1/2, −*z*+3/2; (vii) −*x*+1/2, *y*−1/2, *z*; (viii) −*x*−1/2, *y*−1/2, *z*; (ix) −*x*+2, −*y*+1, −*z*+1; (x) *x*−1/2, −*y*+3/2, −*z*+1; (xi) −*x*, *y*+1/2, −*z*+3/2; (xii) *x*+1/2, *y*, −*z*+3/2; (xiii) *x*+1, *y*, *z*; (xiv) −*x*+1, −*y*+1, −*z*+1.

### Hydrogen-bond geometry (Å, °)

**Table:**

*D*—H···*A*	*D*—H	H···*A*	*D*···*A*	*D*—H···*A*
C3—H3···O3^xi^	0.93	2.60	3.360 (3)	140
C7—H7···O2^x^	0.98	2.56	3.287 (3)	131
C17—H17···O2	0.93	2.55	3.148 (3)	123
C18—H18···O5^ix^	0.93	2.48	3.382 (3)	165

Symmetry codes: (xi) −*x*, *y*+1/2, −*z*+3/2; (x) *x*−1/2, −*y*+3/2, −*z*+1; (ix) −*x*+2, −*y*+1, −*z*+1.

### Table 2 The dihedral angles between the mean planes of the rings (°).

**Table:**

	Ring-2	Ring-3	Ring-4
Ring-1	68.34 (13)	83.04 (13)	3.37 (13)
Ring-2		47.32 (11)	70.49 (10)
Ring-3			83.00 (11)

Ring 1 : N1/C7–C9 β-lactam ring, Ring 2 : C1–C6 benzene ring, Ring 3 : C10–C15 benzene ring, Ring 4 : C16–C21 benzene ring.
